# Rare Gingival Metastasis by Hepatocellular Carcinoma

**DOI:** 10.1155/2017/3192649

**Published:** 2017-03-12

**Authors:** Li-Jun Xue, Xiao-Bei Mao, Jian Geng, Ya-Nan Chen, Qian Wang, Xiao-Yuan Chu

**Affiliations:** Department of Medical Oncology, Jinling Hospital, Nanjing University Clinical School of Medicine, 305 East Zhongshan Road, Nanjing, Jiangsu Province, China

## Abstract

Hepatocellular carcinoma (HCC) uncommonly metastasizes to the gingiva, which always means a poor outcome. We reported a rare HCC case with multiple metastases to gingiva, lungs, and brain. A 60-year-old man was initially diagnosed as HCC with metastases to double lungs. He was subjected to a transarterial chemoembolization (TACE) (5-fluorouracil, 750 mg) and two cycles of intravenous chemotherapy (gemcitabine 1.8 g at days 1 and 8, oxaliplatin 200 mg at day 2, every 4 weeks). However, the volume of liver tumor still increased. A bean-size gingival nodule growing with occasional bleeding was also found. TACE (5-fluorouracil 750 mg, perarubicin 40 mg, cisplatin 20 mg) was performed again and an oral sorafenib therapy (400 mg, twice per day) was adopted. The disease maintained relatively stable for about 6 months until a second obvious progress. The gingival nodule was then palliatively excised and identified as a poorly differentiated metastatic HCC by histopathological examination. Best supportive treatments were made since the performance score was too bad. Finally, cerebral metastases occurred and the patient died of systemic failure. Upon review of previous reports, we discussed risk factors, clinical and pathological characteristics, treatments, and prognosis of gingival metastasis by HCC.

## 1. Introduction

According to recent data, hepatocellular carcinoma (HCC) ranks sixth for cancer incidence and fourth for cancer deaths around the world [1]. Although embolization, radiotherapy, chemotherapy, and novel targeted therapy (sorafenib) have thrown much light, extensive cancer spreading including intrahepatic, abdominal, pulmonary, and cerebral metastases always significantly reduces the overall survival (OS) of patients with advanced HCC [2]. Some uncommon metastases such as those in oral cavity especially indicate a high malignant nature and poorer outcome of HCC [3]. Oral metastatic neoplasms are seldom seen and only reported in the minority of cancer patients, in which metastases to oral soft tissues remain rarer than to jawbones [4–6].

In the present article, we demonstrated an advanced HCC patient with rare metastases to the gingiva besides lungs and brain. Moreover, upon review of previous reports through searching PubMed and Google Scholar databases, we discussed the risk factors, clinical and pathological characteristics, treatments, and prognosis of gingival metastasis by HCC. The used key words included cancer or carcinoma or tumor or neoplasia, liver or hepatic or hepatocellular, metastasis or metastases, and gingiva or mouth or “oral cavity” or “oral mucosa.” Reference lists of all selected references were searched as a secondary source.

## 2. Case Report

**Figure 1:**
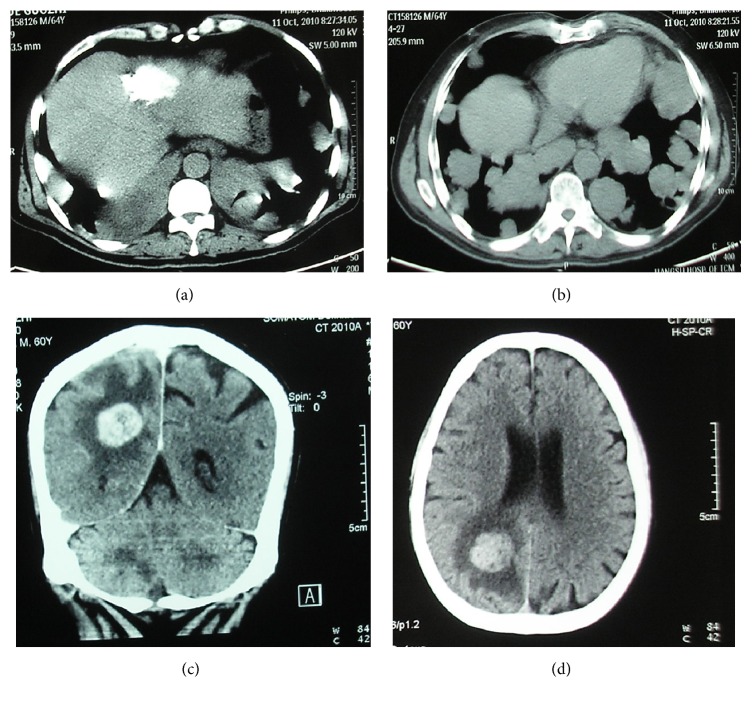
Computerized tomography images of the involved organs. (a) The primary liver mass after the transarterial chemoembolization. (b) Multiple metastases to double lungs. (c and d) Multiple metastases to the brain.

A 60-year-old male with histories of hepatitis B for about 20 years and liver cirrhosis for one year firstly visited a basic hospital with a chief complaint of chronic abdominal pain. A significantly elevated alpha-fetoprotein (AFP) level (2494.2 ng/mL; normal range, <10.9 ng/mL) was revealed. Computerized tomography (CT) scans of abdomen and chest exhibited an about 4 × 3 cm mass in the left lobe of liver and multiple nodules in two lungs (Figures 1(a) and 1(b)). The patient was clinically diagnosed as advanced HCC and subjected to a transarterial chemoembolization (TACE) via the hepatic artery using 750 mg of 5-fluorouracil. At the following month, two cycles of intravenous therapy with gemcitabine (1.8 g, days 1 and 8) and oxaliplatin (200 mg, day 2) were made every 4 weeks. However, the volume of liver tumor still increased and serum AFP level was continuously elevated to as high as 8683.0 ng/mL. A bean-size gingival nodule was also found with mild pain and made discharge of “pus” by puncturing but recurred and grown quickly. TACE using 5-fluorouracil (750 mg), perarubicin (40 mg), and cisplatin (20 mg) was performed again and a sorafenib therapy (400 mg, twice per day) was adopted. The disease then maintained relatively stable for about 6 months until a second obvious progress.

**Figure 2:**
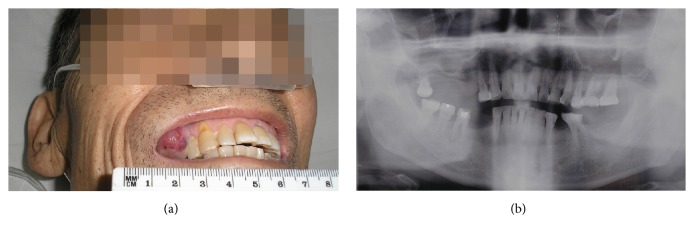
The gingival tumor and jawbone images. (a) A reddish gingival tumor of soft texture, clear margin, and central mucosal ulceration on the right upper maxillary. (b) Radiography without local bony destruction.

**Figure 3:**
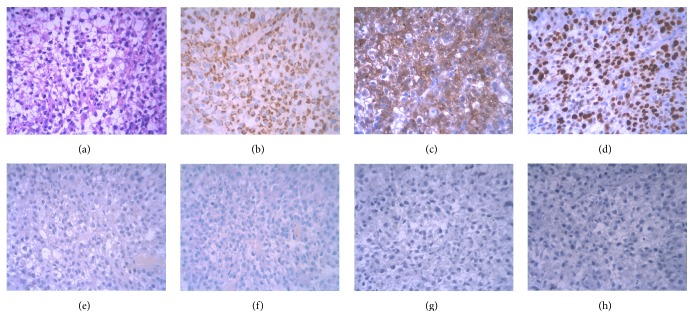
Histopathological staining findings. (a) H&E staining showing high nucleus/cytoplasm ratios and numerous abnormal mitotic divisions in sampled cells. (b–h) Immunohistochemical examinations revealing positive Hep Par 1 and Villin, 80% positive Ki-67, focused weak positive CK 7, and negative AFP, CK 19, and CK 20. Magnification ×200.

When admitted to our hospital, the patient mainly complained of breathlessness, progressive emaciation, serious notalgia, and right upper quadrant pain. Laboratory tests showed anemia (hemoglobin 91 g/L), thrombocytopenia (platelet count 97 × 10^9^/L), hypoproteinemia (albumin 33.4 g/L), and elevated serum levels of AFP (4325.0 ng/mL), aspartate aminotransferase (66 U/L; normal range, <50 U/L), lactate dehydrogenase (296 U/L; normal range, 60–240 U/L), direct (9.4 *μ*mol/L; normal range, ≤6.8 *μ*mol/L), and indirect bilirubin (13.2 *μ*mol/L; normal range, ≤12.2 *μ*mol/L), with basically normal blood clotting function. Physical examination showed a reddish 2 × 2 cm gingival nodule on the alveolar mucosa distal to the upper right first premolar, which was of soft texture, clear margin, and central mucosal ulceration, with swelling, mild pain, and occasional bleeding but no bony destruction by radiography (Figures 2(a) and 2(b)). A dentist made a palliative excision of the nodule under local infiltration anesthesia and found much rotten fish-like discharge, which was confirmed as poorly differentiated carcinoma by histopathological and immunohistochemical (IHC) examinations, tending to be metastasized by HCC with positive hepatocyte paraffin 1 (Hep Par 1) and Villin, 80% positive Ki-67, focused weak positive cytokeratin 7 (CK 7), and negative AFP, CK 19, and CK 20 (Figures 3(a)–3(h)). Nevertheless, the gingival nodule recurred the next day. Gradually, the patient suffered from frequent headache, projectile vomiting, dysphagia, hydroposia bucking, and numbness of limbs, although symptoms of pain and breathlessness were improved. CT scan of the head showed metastasized tumors (about 2.5 cm in diameter) with significant peritumoral edema in the right cerebellum and parietal and occipital brain lobes (Figures 1(c) and 1(d)). Best supportive treatments were made but he finally died of systemic failure.

## 3. Discussion

The metastasis of HCC to gingiva usually relates to extensive tumor spreading and occurs relatively late. Besides its low incidence rate, the easily ignored clinical symptoms and signs also contribute to the rareness of pathologically confirmed gingival metastasis in advanced HCC patients.

Metastatic gingival malignancy usually exhibited as a soft hyperemic nodule, with or without local pain [17]. Regional swelling, ulceration, and bleeding are much common like in our case and sometimes present as the earliest symptoms, to which enough attention should be paid [3, 11, 12, 17, 19]. Notably, gingival bleeding usually does not but perhaps relates to abnormal coagulation parameters due to defective liver functions, especially when the symptom is too severe to be controlled [12, 20]. Among all clinical signs, rapid recurrence, progressive growth, and invalid antibiotic therapy of an unknown gingival mass in HCC patients like this case will be the most important signal for differential diagnosis [12, 21].

In advanced HCC cases with a big oral tumor or synchronous jawbone metastasis, however, it may be difficult to discriminate whether a gingival neoplasm originates from the secondary invasion of metastasized jaw tumor. Anyway, a timely biopsy is firstly suggested to exclude a metastatic oral tumor in cancer sufferers, which is easily confused with inflammatory or benign reactive lesions [12, 17]. IHC examinations help to identify both the nature and origin of gingival neoplasm. Hep Par 1 positivity in the specimen often sensitively and specifically determinates the hepatic origin and CK 7, CK 19, and CK 20 negativities favor the confirmation of malignant nature [22]. AFP staining aids in the diagnosis of metastatic HCC with a positivity rate at approximately 30%, rather than other adenocarcinomas [23]. In this case, the morphologic features in sampled cells with high nucleus/cytoplasm ratios and numerous abnormal mitotic divisions by hematoxylin and eosin (H & E) staining, together with positive Hep Par 1, focused weak positive CK 7, and negative CK 19 and CK 20 by IHC, have established the definitive diagnosis of gingival metastatic HCC. As reported, the progenitor cell marker CK 19 is significantly associated with poor survival but only has a positivity rate of 18.2% in HCC patients [24]. Positive Villin and Ki-67 indicate the hepatic origin and cellular proliferation extent, respectively, and support the HCC diagnosis, although AFP is negatively stained.

Among this series and other published cases in Korean or Japanese languages, more patients were from Asian areas including Japan, China, Korea, and Indonesia [3, 7–18, 25–29]. It seems that Asia should be the specific region with highest morbidity rate of HCC-metastatic gingival tumor, which is probably attributed to the geographic epidemiological characteristic of HCC [11, 12, 17]. As reported, the mean age and male-to-female sex ratio are 42 years and 2 : 1, respectively, in patients with oral soft tissue metastases by various primary carcinomas [6]. In the present series, gingival metastasis by HCC tends to present more often in male patients with higher male-to-female ratio of 6 : 1 (12/2) and median age of 60 (range, 44–78) years, which may correspond with the male predominance of HCC. In addition, HCC nearly always spreads to the gingiva of upper jaw rather than to that of lower jaw, nevertheless, the reasons for which remains unknown [17]. Other metastases besides within the liver and to the gingiva usually include lungs, brain, regional lymph nodes, adrenals, and musculoskeletal system (Table 1).

It is still unclear why just very few of HCC patients show the tendency to spread to the gingiva. Pathogenesis of this special metastasis is thought to be associated with oral inflammation that possibly attracts migration and adhesion of cancer cells to the gingiva, in which some inflammatory molecules might play key roles [4]. Others ever proposed that the localized slowing of blood flow contribute to gingival metastasis by favoring the fall-out of malignant cells [30, 31]. The hematogenous route through portal vessels is the preferred mode for oral metastasis; however, metastatic pulmonary tumors are not found in some cases as expected [17]. In such instance, the bypassing of pulmonary circulation infiltration through a candidate pathway of valveless vertebral venous plexus (Batson's plexus) has been theoretically inferred to be an explanation, which remains to be anatomically or experimentally verified [31, 32]. Moreover, the regional lymphatic vessel is likewise a possible predominate route for gingival metastasis by HCC [9]. In this series, liver cirrhosis presents in about 50% of HCC patients with metastatic gingival tumor (data not shown). Herein, we cautiously propose an assumption that the changing hemodynamics due to esophageal varices be one of potential pathways for oral metastases particularly in those HCC patients with liver cirrhosis of broken compensation, which still needs further confirmation. Notably, additional reason for the rare occurrence of gingival metastasis may be that most cases are probably not reported.

In most instances, both the overall and truncated survival (from onset of oral metastatic tumor to death) in HCC patients with oral metastasis are very poor, with the average time being about 7 months and 21 weeks (range, 2 weeks to 2 years), respectively [3, 6, 12, 17]. Up to now, the survival of gingival metastasis by HCC is not very clear because of the rareness and scattering of such cases, which are always published without more detailed and systematic description. According to limited series with survival data in English literatures, the median time of overall and truncated survival for HCC patients with gingival metastasis is about 11.5 months (range, 2 to 92 months) and 3.5 months (range, 1 to 15 months), respectively (Table 1). Herein, many cases, which are individually reported or included in systematic reviews but without complete survival data or accurate description of gingival metastasis, have been excluded [4–6, 19, 29–31, 33–35]. A diagnosed case of gingival metastasis with synchronic HCC and primary prostate cancer has also been excluded because the survival may be markedly influenced by the second malignancy [36]. Notably, the resection of oral metastatic neoplasm might help to better the survival of certain patients although it seems to be palliative in most cases [9, 15–17]. Enough recognition, early diagnosis, and appropriate management of gingival metastasis may help to improve the outcome of this special subgroup of HCC. Anyway, further retrospective or even prospective studies with a larger size of patients are needed.

## Figures and Tables

**Table 1:** Cases of gingival metastasis by hepatocellular carcinoma with survival data in English literatures.

Case number	Sex/age	Gingival tumor as first sign	Cancer differentiation	Upper/lower jaw	Distant metastasis besides gingival	Therapy	Truncated/overall survival^*∗*^	Reference number
1	M/56	Yes	NA	Upper	Lungs, pancreas, adrenal, LNs	NA	2/2 months	[7]

2	M/51	Yes	Undifferentiation	Upper	Lung, LNs, skin, peritoneum	Resection of gingival tumor	2/2 months	[8]

3	M/52	No	High differentiation	Lower	Lungs	Hepatectomy, resection of gingival tumor	15/39 months	[9]

4	M/56	No	NA	Upper	None	Resection of gingival tumor	1/6 months	[10]

5	M/64	No	NA	Upper	Lungs, adrenals, LNs	TACE, resection of gingival tumor	1/22 months	[11]

6	F/78	Yes	Moderate differentiation	Upper	Skull, lumbar vertebrae	Palliative excision of gingival tumor	4/4 months	[12]

7	M/66	Yes	NA	Lower	None	Resection of gingival tumor	5/5 months	[13]

8	M/44	No	NA	Upper	Nasal cavity, bone	Chemotherapy	>3/24 months	[14]

9	M/70	Yes	Moderate differentiation	Upper/lower	Lungs, brain	Resection of gingival tumor, TACE	8/8 months	[15]

10	M/55	No	NA	Lower	Mandible and iliac bones, ribs, scapule, pleura, brain	Liver tumor alcoholization, TACE, segmental resection of left mandible	>8/92 months	[16]

11	M/65	No	NA	Upper	None	Resection of gingival tumor, chemotherapy	8/15 months	[17]

12	M/60	No	Moderate differentiation	Lower	Lung, skin of multiple sites	Partial hepatectomy and TACE	6/38 months	[3]

13	F/72	No	Moderate differentiation	Upper	Cardiac muscle, abdominal LN	TACE, radiotherapy in gingival tumor	1/10 months	[18]

14	M/60	No	Poor differentiation	Upper	Lungs, brain	Chemotherapy, TACE, sorafenib, palliative resection of gingival tumor	2/13 months	Present case

M, male; F, female; NA, not available; LN, lymph node; TACE, transarterial chemoembolization.

^*∗*^Truncated survival, the period from onset of oral metastasis to death.
